# A Novel Strategy for Attenuating Opioid Withdrawal in Neonates

**DOI:** 10.4172/2155-6105.1000291

**Published:** 2016-08-11

**Authors:** Giovanni C Santoro, Samarth Shukla, Krishna Patel, Jakub Kaczmarzyk, Stergiani Agorastos, Sandra Scherrer, Yoon Young Choi, Christina Veith, Joseph Carrion, Rebecca Silverman, Danielle Mullin, Mohamed Ahmed, Wynne K Schiffer, Jonathan D Brodie, Stephen L Dewey

**Affiliations:** 1 Center for Neurosciences, Laboratory for Molecular and Behavioural Neuroimaging, Feinstein Institute for Medical Research, Manhasset, NY, USA; 2 Division of Neonatal-Perinatal Medicine, Cohen Children’s Medical Center of NY, New Hyde Park, NY, USA; 3 Department of Molecular Medicine, Hofstra North Shore-LIJ School of Medicine, Hempstead, NY, USA; 4 Department of Neurology, N. Bud Grossman Center for Memory Research and Care, University of Minnesota, Minneapolis, MN, USA; 5 Psychiatry Department, New York University School of Medicine, NY, USA

**Keywords:** GVG, Neonatal abstinence syndrome, GABA, Vigabatrin, PET imaging, Withdrawal behaviour, Prenatal intervention

## Abstract

The rate of Neonatal Abstinence Syndrome (NAS) has drastically increased over the past decade. The average hospital expense per NAS patient has tripled, while the number of babies born to opioid-dependent mothers has increased to 5 in 1000 births. Current treatment options are limited to opioid replacement and tapering. Consequently, we examined the efficacy of prenatal, low-dose and short-term vigabatrin (γ-vinyl GABA, GVG) exposure for attenuating these symptoms as well as the metabolic changes observed in the brains of these animals upon reaching adolescence. Pregnant Sprague-Dawley rats were treated in one of four ways: 1) saline; 2) morphine alone; 3) morphine+GVG at 25 mg/kg; 4) morphine+GVG at 50 mg/kg. Morphine was administered throughout gestation, while GVG administration occurred only during the last 5 days of gestation. On post-natal day 1, naloxone-induced withdrawal behaviours were recorded in order to obtain a gross behaviour score. Approximately 28 days following birth, ^18^FDG microPET scans were obtained on these same animals (Groups 1, 2, and 4). Morphine-treated neonates demonstrated significantly higher withdrawal scores than saline controls. However, GVG at 50 but not 25 mg/kg/day significantly attenuated them. Upon reaching adolescence, morphine treated animals showed regionally specific changes in ^18^FDG uptake. Again, prenatal GVG exposure blocked them. These data demonstrate that low-dose, short-term prenatal GVG administration blocks naloxone-induced withdrawal in neonates. Taken together, these preliminary findings suggest that GVG may provide an alternative and long-lasting pharmacologic approach for the management of neonatal and adolescent symptoms associated with NAS.

## Introduction

Every hour one baby is born in the United States suffering from Neonatal Abstinence Syndrome (NAS), a drug withdrawal disorder caused by gestational opioid abuse. Symptoms include autonomic dysregulation, seizures, difficulty feeding, and low birth weight. Between 2000 and 2012, the incidence of NAS significantly increased from 1.20 to 5.80 per 1000 hospital births per year (p<0.001). Simultaneously, opioid use increased 5 times [1–3]. While morphine, methadone, and buprenorphine replacement therapies are commonly y used to treat NAS, these drugs produce dependence themselves [4], frequently require prolonged treatment [5–7] and cause further neurodevelopmental and cognitive delays [8–11]. One review described neurodevelopmental outcomes of infants exposed to opioids in utero. These data strongly suggest that infants born to opioid-dependent mothers are at high risk of cognitive and motor delay, persisting at least into the pre-school years [12]. Additionally, preliminary MRI data suggest altered maturation of connective neural tracts within the first six weeks of life, following in utero opioid exposure but these findings have yet to be correlated with longer term outcome [13]. Thus, the development of an effective, non-addictive, non-narcotic and cost-effective treatment for NAS has become a national healthcare concern [14,15].

There is an increased interest in selectively targeting γ-aminobutyric acid (GABA) for the treatment of opioid withdrawal [16–18]. We are particularly interested in the GABA mimetic compound, γ-vinyl GABA (GVG, vigabatrin, marketed as Sabril^®^), a non-addictive, non-narcotic, irreversible inhibitor of GABA-transaminase (GABA-T). The U.S. Food and Drug Administration (FDA) recently approved GVG for an indication of infantile spasms and as an adjunct therapy for refractory complex partial seizures. Further, a large body of preclinical work has demonstrated that GVG blocks heroin self-administration [19] and inhibits heroin, cocaine, amphetamine/methamphetamine and alcohol-induced increases in extracellular dopamine [20]. Finally, a series of three clinical trials using GVG demonstrated its and safety for the treatment of cocaine addiction in adults [21–23]. While a more recent US clinical trial failed to reproduce these findings, ongoing investigations into these data are examining differences between them. More specifically, differences in subject selection, disease severity and length of substance dependence, motivation to eliminate use and the style and setting of treatment are important contributors to the success or failure of GVG, or any drug for that matter, in substance abuse treatment.

GVG is FDA approved for use during pregnancy. Case studies have demonstrated no obvious side effects of in utero exposure at low doses [24,25]. GVG crosses the placenta by simple diffusion through a hydrophilic pathway. Its elimination half-life in epilepsy patients is reported to be between 5.3 and 7.4 h, while its biologic half-life may be longer due to its irreversible inhibition of GABA-T, which can take up to 6 days to resynthesize following drug cessation [11,26]. Even though GVG is not protein bound, cerebrospinal concentrations were found to be only 10% of the plasma concentration 6 h following a single oral dose [27]. Based on these findings and the favourable pharmacokinetic profile of GVG, we hypothesized that low-dose, short-term, prenatal GVG exposure would effectively attenuate naloxone-induced withdrawal in neonatal rats exposed to morphine throughout gestation.

## Materials and Method

This study was conducted in strict accordance with the recommendations in the Guide for the Care and Use of Laboratory Animals of the National Institutes of Health. The protocol was approved by the Institutional Animal Care and Use Committee (IACUC).

**Figure 1:**
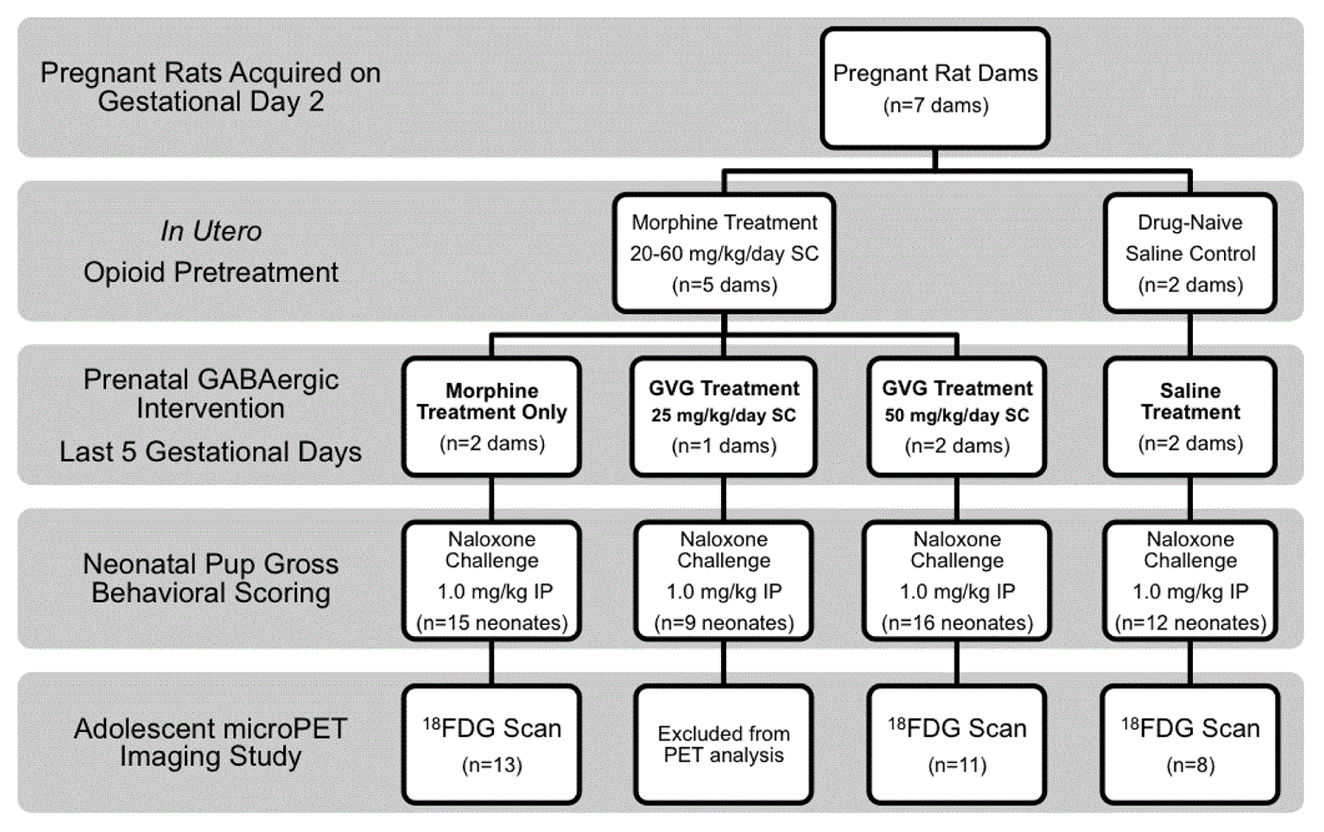
Study overview and timeline, Morphine was administered to pregnant dams in escalating dose (20–60 mg/kg/day) for the first 6 days and 60 mg/kg/day each day until parturition. Morphine +GVG groups received morphine in the same dose as morphine only groups. The morphine+GVG25 group received GVG at 25 mg/kg/day and the morphine+GVG50 group received GVG at 50 mg/kg/day. GVG was administered only for the last 5 days of gestation.

Timed-pregnant Sprague-Dawley rats were acquired from Taconic Farms and arrived on gestational day (GD)2. Upon arrival, dams received one of 4 treatments: 1) saline; 2) morphine alone; 3) morphine +GVG at 25 mg/kg (morphine+GVG25); 4) morphine +GVG at 50 mg/kg (morphine+GVG50). Morphine groups received an escalating dose (20–60 mg/kg, for 6 days) and then 60 mg/kg/day until parturition (Figure 1). This escalating dosing scheduled was implemented to prevent morphine-induced infant loss. On the day of parturition, litters were randomly culled to 9 pups [28] and transferred to surrogates to ensure adequate nutrition. Weights were recorded daily from PND 0-21.

On postnatal day (PND) 1, neonates received an acute challenge of naloxone hydrochloride (1.0 mg/kg, intraperitoneally (IP)). Immediately following this challenge, animals were returned to their specially-designed, noise-attenuating, thermo-regulated environments with overhead cameras. Behaviours were recorded via video cameras from 15 min pre-naloxone administration to 45 min post-naloxone administration. Naloxone administration rapidly induced withdrawal. Videos were time sampled at 15 min intervals and were scored for frequency of locomotion, rolling, curling, and stretching [29,30], by three trained raters blinded to the treatment condition.

Three raters tallied the frequency of each behaviour. Rater’s scores were averaged to get an Individual Behaviour Score (IBS) for each behaviour. Finally, the IBS_locomotion_, IBS_rolling_, IBS_curling_, IBS_stretching_ for each animal were summed to get a Gross Behaviour Score (GBS) for each animal. The GBS was used to represent the intensity of withdrawal, and was compared between treatment groups. After ensuring that the data were approximately normally distributed [31–33] parametric statistical analysis was utilized. A multivariate ANOVA was carried out. A significant ANOVA prompted post hoc Fischer LSD tests. The experimental unit (EU) in these analyses was the neonate. Concern over a potential litter effect, prompted additional analyses using litters as the EU [34,35]. The average litter GBS [36] was acquired and analysed using a series of repeated-measures ANOVAs to verify that the observed effects of treatment seen in the first analysis were not due to Type-I error.

At 4 weeks following birth (adolescence), 33 pups (saline group=9 pups, morphine group=13 pups, morphine+GVG50 group=11 pups) were randomly selected and anesthetized using ketamine/xylazine according to a standard animal anaesthesia protocol. Micro-positron emission tomography (microPET) scans were obtained using [^18^F[Fluoro-2-deoxy-2-D-glucose (^18^FDG) in a Siemen’s Inveon tomograph. All emission images were corrected for attenuation. MicroPET images were analysed for regional increases and decreases in ^18^FDG uptake using PMOD (PMOD Technologies Ltd., Zurich, Switzerland). Images from saline, morphine and morphine+GVG50 groups were compared using SPM 5.0 (Statistical Parametric Mapping, MATLAB, The Mathworks, Inc., USA).

## Results

**Figure 2:**
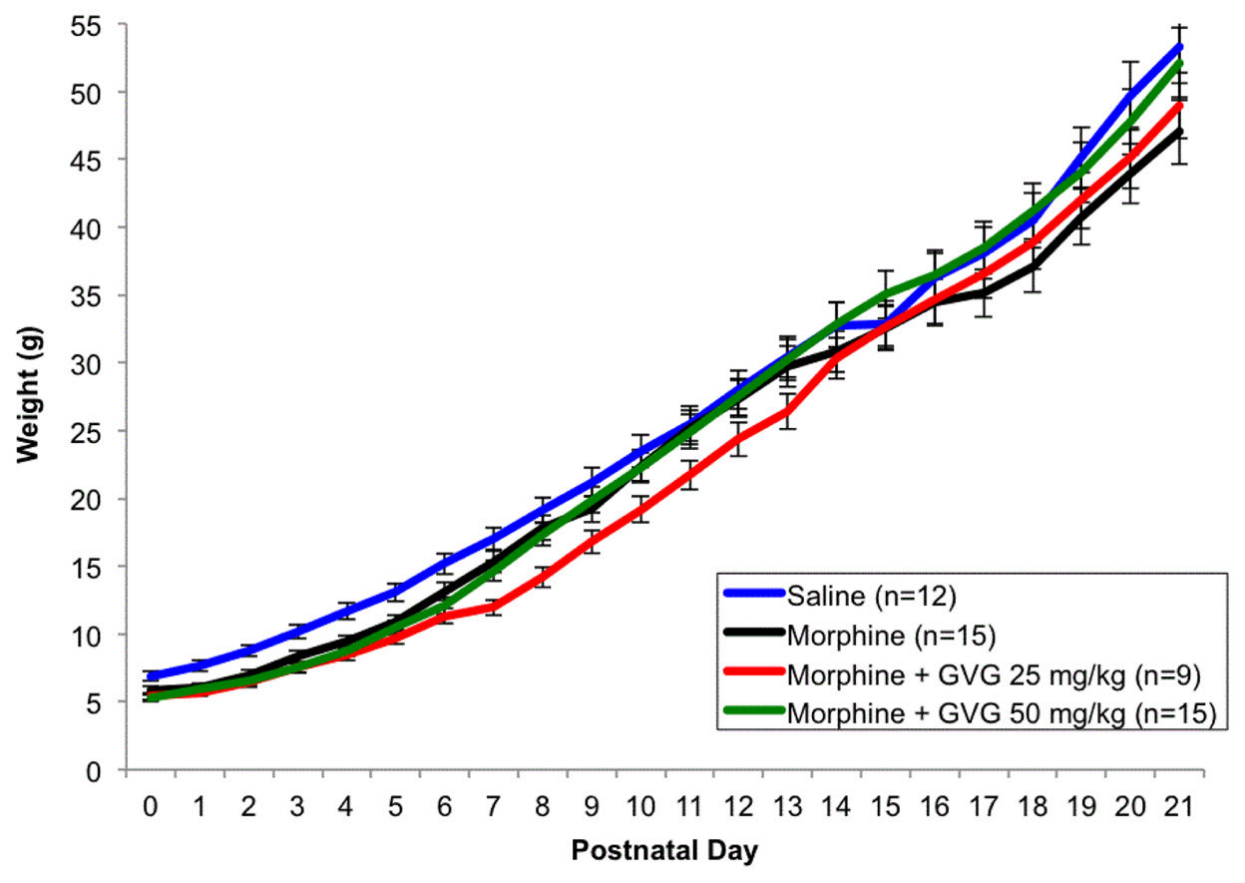
Average pup weight gain over time, Average daily pup weight up to PND 21. Birth weights from morphine, morphine +GVG25 and morphine+GVG50 treatment groups were significantly lower than control pups (p<0.01). However, by PND 21, weights of the morphine+GVG25 (p=0.90) and morphine +GVG50 groups (p=0.49) returned to control values while pup weights exposed to morphine alone did not (p=0.01).

Morphine alone or in combination with GVG did not alter litter size. However, there were significant differences in birth weights and postnatal weight gain between groups (Figure 2).

**Figure 3:**
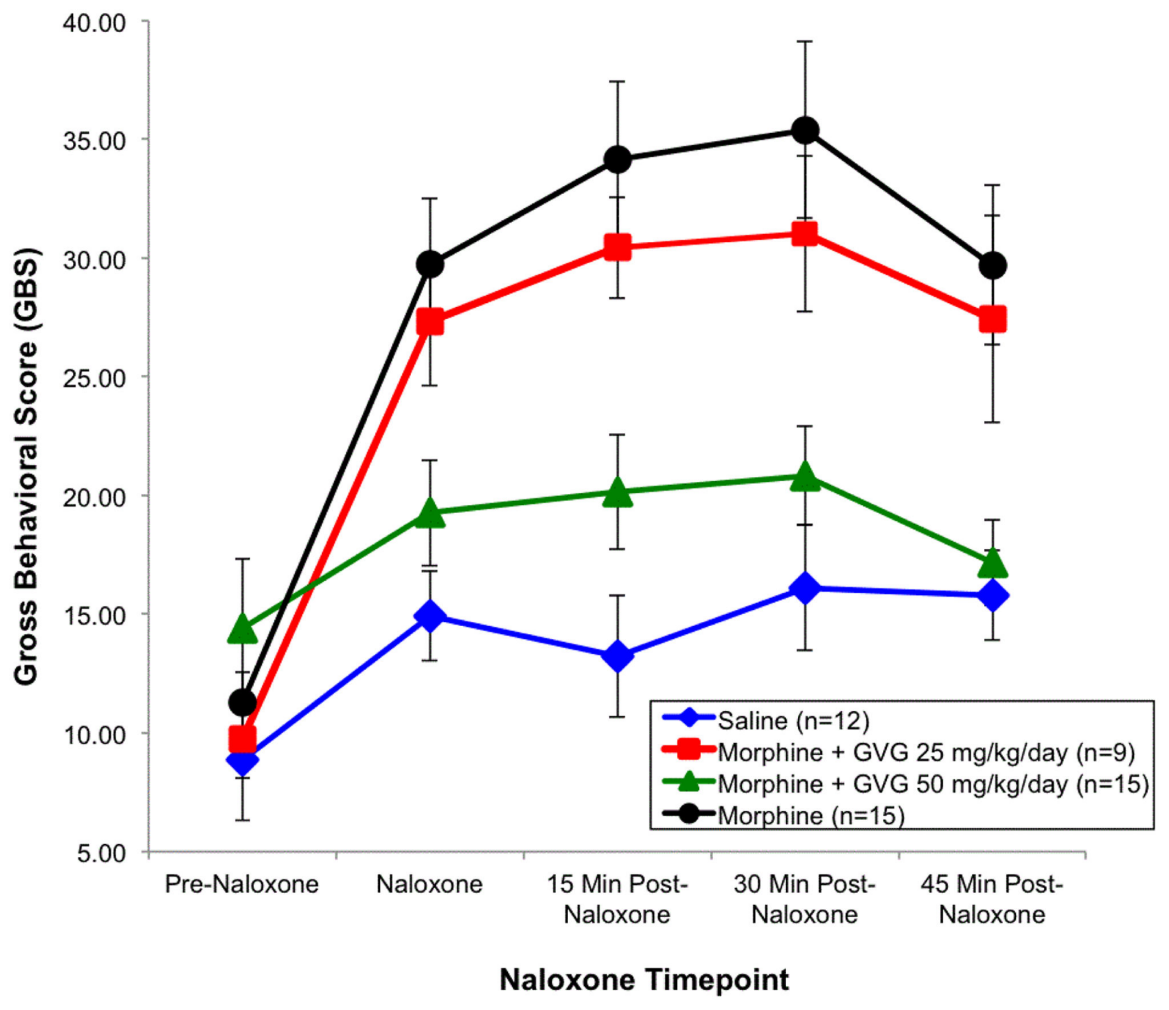
Withdrawal behaviour vs. naloxone time point, Intensity of withdrawal before and after naloxone administration.

In the first series of behavioural analyses, using the neonate as the EU, a significant effect of treatment was observed (p=0.003). Subsequent LSD post hoc tests were carried out and revealed that there were no significant differences in GBS between the treatment groups at the pre-naloxone time point. However, there were differences at 15, 30 and 45 min following acute naloxone administration. Neonates exposed to morphine alone had significantly higher GBS scores than saline animals at the naloxone time point (p=0.0005), 15 min time point (p=0.0005), 30 min time point (p=0.0005), as well as the 45 min time point (p=0.001). The morphine+GVG50 treatment group did not differ from the saline group at the pre-naloxone (p=0.090), naloxone (p=0.208), 15 min (p=0.080), 30 min (p=0.266) and 45 min (p=0.730) time points. The morphine+GVG50 group did, however, from the morphine group at the naloxone (p=0.002), 15 min (p=0.0005), 30 min (p=0.001), and 45 min (p=0.002) time points. Subsequent inspection of the means indicated that the saline and morphine +GVG50 subjects had similar behavioural scores. The morphine +GVG25 group did not differ significantly from any other group at the pre-naloxone time point, but did have significantly higher GBS than saline at the naloxone (p=0.002), 15 min (p=0.005), 30 min (p=0.003) and 45 min time points (p=0.013) and morphine+GVG50 at the same time points (p=0.033, 0.018, 0.030, 0.021 respectively) (Figure 3).

In short, the morphine and the morphine+GVG25 groups exhibited significantly higher GBS than either the saline or morphine+GVG50 groups. That is, the morphine+GVG25 group did not differ significantly from the morphine alone group. However, the morphine +GVG50 group displayed a similar degree of gross behavioural activity effect as the saline group. To rule out a potential due to litter, a subsequent analysis was carried out using litter means as the EU.

A repeated measures ANOVA carried out on the GBS litter averages revealed that there was a significant effect of behavioural time point (p=0.009). This suggests that on average, there were differences in average GBS at several time points. In general, certain litters had more activity on average at each time point. Inspection of the means revealed that saline and morphine+GVG50 dams had the lowest GBS, morphine had the highest, and morphine+GVG25 fell somewhere in between. Since it is theoretically possible that these differences could be due handling during naloxone treatment and post-injection hyperactivity, the same analysis was carried out after removing morphine+GVG25 and morphine dams. Comparison of the GBS of saline and morphine+GVG50 dams alone revealed no significant differences at any time points (p=0.118). Similar comparisons looking solely at morphine and morphine+GVG25 groups indicate that these groups do differ at various time points (p=0.037) and that morphine +GVG25 and morphine+GVG50 do not differ when compared (p=0.190). This differs from our previous analysis, and could indicate a potential subtherapeutic effect of GVG (25 mg/kg). Although this second analysis was carried out to rule out a potential litter effects, we do acknowledge that when averaging litters into experimental units, there is a reduction in sample size, which could influence power.

**Figure 4:**
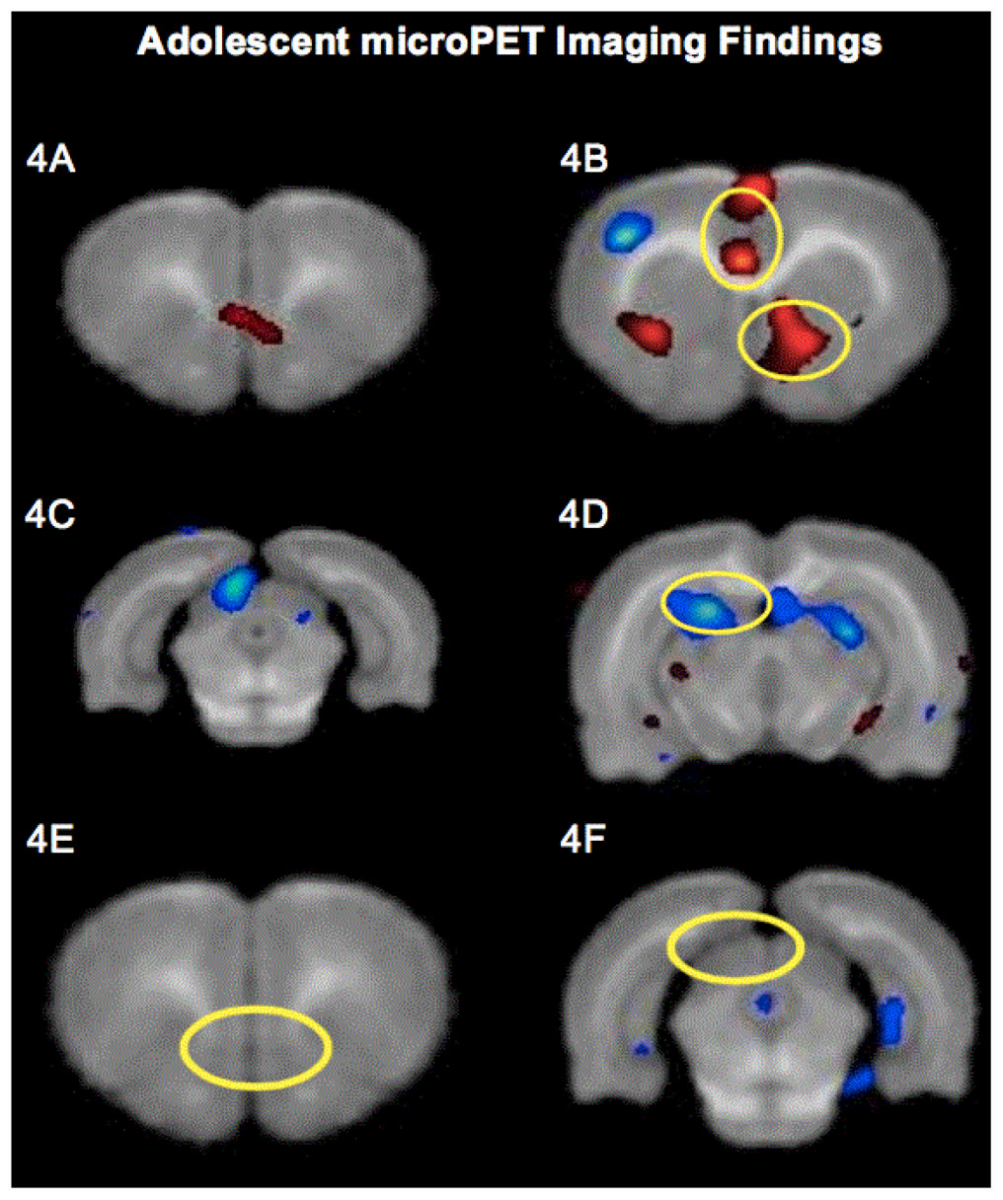
Adolescent microPET ^18^FDG Imaging Findings, PET images showing increased ^18^FDG uptake in infralimbic cortex (A) as well as nucleus accumbens and cingulate cortex; (B) of morphine group as compared to saline group (p<0.01) PET images showing decreased ^18^FDG uptake in superior colliculus; (C) and hippocampus (D) of morphine group as compared to saline group (p<0.01) PET images showing unaltered ^18^FDG uptake in infralimbic cortex; (E) and superior colliculus; (F) of morphine +GVG50 group as compared to morphine group (p>0.01).

Inter-group comparisons of microPET images obtained once these animals reached adolescence demonstrated that the morphine group had increased ^18^FDG uptake in the nucleus accumbens, cingulate cortex and infralimbic cortex. Further, the morphine group had decreased ^18^FDG uptake in the superior colliculi and parts of the hippocampus. However, the infralimbic cortex and superior colliculi were unaffected in the morphine+GVG50 group (Figure 4).

## Conclusion

Taken together, these demonstrat that neonates born to morphine-treated dams exhibited marked withdrawal behaviours following an acute naloxone challenge. Administration of GVG at 25 mg/kg/day during the last 5 days of gestation failed to alter this behaviour. However, when administered at 50 mg/kg/day for the same period of time, GVG reduced the GBS to control levels. Since GVG at 25 mg/kg/day was ineffective at reducing withdrawal behaviour, this group was excluded from subsequent microPET imaging.

Analyses of microPET images demonstrated that prenatal morphine exposure produced changes in brain metabolism that persisted into adolescence. Specifically, these changes were noted in the nucleus accumbens, cingulate cortex, infralimbic cortex, superior colliculi, and parts of the hippocampus. These regions have been associated with reward modulation, learning and reinforcement, fear inhibition, vision, attention shifting, and memory with spatial navigation. Prenatal GABAergic intervention eliminated these metabolic changes in the infralimbic cortex and the superior colliculi.

## Discussion

These data suggest that a low-dose pharmacotherapeutic strategy selectively targeting the GABAergic system late in gestation may effectively attenuate or even eliminate acute opioid withdrawal behaviours in neonates. Further, this approach also appears to selectively diminish alterations in brain glucose metabolism in regions associated with fear inhibition, vision, and attention shifts that persist into adolescence. With respect to the clinical implications of these findings, it should be noted that in rats, it is generally accepted that the first two human trimester equivalents occur in utero, between GD 1-21, while the third human trimester equivalent occurs ex utero, from PND 1-7 [30,37]. Thus, in the present study, prenatal GVG treatment occurred during the human equivalent of the terminus of the second trimester.

In our previous studies, GVG administered throughout gestation (GD 2-21) at doses of 150 and 300 mg/kg/day resulted in reduced litter sizes. These doses are within a range previously shown to produce fetal anomalies and growth retardation [38]. Therefore, due to potential teratogenic properties of GVG, the dosing schedule selected for this specifically study was designed to be both low in dose and short in duration. It is important to note that there were no differences in pregnancy weight gain of the dam, litter size, or neonatal weight gain between saline and morphine+GVG groups.

Opioid withdrawal is spontaneous and varies based on drug half-life. For example, heroin withdrawal begins around 8–12 h, peaks between 36–72 h and can last up to two weeks [39–41]. Due to this marked variability associated with spontaneous withdrawal, behavioural scoring can be especially difficult and inconsistent. For these reasons, we employed a more consistent approach in the service of establishing a preclinical animal model that may be used in the subsequent development of new treatment strategies for NAS. Specifically, using an acute dose of the full agonist, naloxone, withdrawal behaviours occur in a quicker, more intense, and most importantly, more reproducible manner [30,42–44]. That is, this acute precipitated design was chosen to ensure that opioid withdrawal behaviour could be recorded and scored within a timeframe that is considerably shorter and far more consistent.

Smith and colleagues [45,46] conducted magnetic resonance spectroscopy studies in children who had been exposed to cocaine or methamphetamine prenatally. They found that their total brain creatinine levels were elevated, suggesting abnormalities in energy metabolism. In the present study, we used microPET imaging to identify brain regions in adolescent animals that experienced altered metabolism as a consequence of prenatal opioid exposure.

Our findings are consistent with adult studies using related drugs for the treatment of opioid withdrawal. GABApentin (Neurotin^®^), a GABA analog, effectively reduced withdrawal symptoms in opioid-dependent patients undergoing methadone-assisted detoxification [16]. Other GABA agonists including carbamazepine, topiramate, tiagabine, and baclofen have also proven effective at reducing withdrawal symptoms associated with opioid exposure [17]. In a single case report, a 43 year old patient suffering from opioid withdrawal was able to control withdrawal symptoms using 300 mg/day of Pregabalin (Lyrica^®^), a GABA-mimetic drug. Pregabalin binds to the α2δ subunit of voltage-gated calcium channels, thereby inhibiting the release of excitatory neurotransmitters including glutamate, norepinephrine, substance P and calcitonin gene-related peptide [18]. These data highlight the clinical importance of noradrenergic and glutamatergic signaling in the regulation of symptoms associated with opioid withdrawal [47].

Pregabalin, GABApentin, and other GABA agonists, potentiate the inhibition of these two excitatory neurotransmitter systems making them useful candidates to alleviate neuronal hyperexcitatory states, such as opioid withdrawal [16]. Furthermore, in prepubescent mice, baclofen prevented the expression of naloxone-induced morphine withdrawal signs. These effects were greater in males than females and were secondary to a regionally specific increase in μ-opioid receptor binding observed in males only [48]. Taken together, these findings suggest that GVG may be working by either potentiating inhibitory control over these excitatory pathways or by preventing μ-opioid receptor downregulation secondary to chronic opioid exposure. While the mechanism of GVG is likely related to that of the other GABAmimetic compounds discussed, there is an advantage that GVG might have over these compounds. Namely, our novel approach utilizes an enzyme-mediated mechanism of action.

Pregabalin, GABApentin, and other agonists exert their effect on the GABA receptor complex or transporter. GVG, alternatively, acts as an irreversible inhibitor of GABA-transaminase (GABA-T), which is responsible for the catabolism of GABA. GVG administration therefore leads to increased brain GABA [49,50], which likely modulates excitatory hyperactivity and consequently reduces morbidity and mortality in animals born to opioid-dependent dams. Furthermore, since GVG does not directly compete for opioid or any other receptors, it is less likely to produce dependence, tolerance, or withdrawal [51–53].

Abdulrazzaq et al. reported that in pregnant mice, peak levels of GVG occurred in the placenta and embryo about 3.5 h following an acute injection and decreased remarkably within 6 h [37]. In the present study, the mean time between the last dose of GVG administered to pregnant dams and the acute naloxone challenge administered to their offspring was approximately 44 h. Together, these data suggest that GVG concentrations in these neonates, at the time of their naloxone challenge, would be minimal if at all measureable.

Although GVG has a very short elimination half-life, it has been found to have a very long biologic effect [26]. This is likely related to the normalization of GABA-T levels, which have been shown to take up to 6 days to return to baseline after treatment cessation [49]. These data in combination with the pharmacokinetic profile of GVG eliminate the need for continuous administration to a neonate. By allowing the developing brain to leverage endogenous plasticity, GVG might prevent the signs and symptoms of opioid withdrawal. The advantage of treating dams immediately prior to parturition is that it precludes withdrawal. If treatment were given postpartum, one would expect that neonatal opioid receptors would already be up-regulated and occupied. Finally, while there seems to be obvious advantages to administering low-dose GVG, an effective, non-addictive, and non-narcotic drug prior to the emergence of NAS, we recognize that it may be possible to intervene at other times with other GABAergic drugs in the perinatal continuum.

## Abbreviations

^18^FDG: [^18^F]Fluoro-2-deoxy-2-D-glucose
EU: Experimental Unit
GABA-T: GABA-Transaminase
GBS: Gross Behaviour Score
GVG: Vigabatrin, γ-vinyl GABA, Sabril^®^
IBS: Individual Behaviour Score
IP: Intra Peritoneal
microPET: Micro Positron Emission Tomography
Morphine+GVG25, Treatment group: Morphine (escalating, Figure 1)+GVG (25 mg/kg)
Morphine+GVG50, Treatment group: Morphine (escalating, Figure 1)+GVG (50 mg/kg)
NAS: Neonatal Abstinence Syndrome
PMOD: PMOD Technologies Ltd
Zurich: Switzerland
PND: Postnatal Day
SPM: Statistical and Parametric Mapping

## References

[1] Wendell AD (2013). Overview and epidemiology of substance abuse in pregnancy. Clin Obstet Gynecol.

[2] O’Donnell M, Nassar N, Leonard H, Hagan R, Mathews R (2009). Increasing prevalence of neonatal withdrawal syndrome: Population study of maternal factors and child protection involvement. Pediatrics.

[3] Kandall SR, Albin S, Lowinson J, Berle B, Eidelman AI (1976). Differential effects of maternal heroin and methadone use on birthweight. Pediatrics.

[4] Bio LL, Siu A, Poon CY (2011). Update on the pharmacologic management of neonatal abstinence syndrome. J Perinatol.

[5] Hudak ML, Tan RC, Committee on drugs; committee on fetus and new-born; American Academy of Pediatrics (2012). Neonatal drug withdrawal. Pediatrics.

[6] Kraft WK, Dysart K, Greenspan JS, Gibson E, Kaltenbach K (2011). Revised dose schema of sublingual buprenorphine in the treatment of the neonatal opioid abstinence syndrome. Addiction.

[7] Lainwala S, Brown ER, Weinschenk NP, Blackwell MT, Hagadorn JI (2005). A retrospective study of length of hospital stay in infants treated for neonatal abstinence syndrome with methadone versus oral morphine preparations. Adv Neonatal Care.

[8] Behnke M, Smith VC, Committee on Substance abuse; Committee on fetus and new-born (2013). Prenatal substance abuse: Short- and long-term effects on the exposed fetus. Pediatrics.

[9] Irner TB (2012). Substance exposure in utero and developmental consequences in adolescence: A systematic review. Child Neuropsychol.

[10] Hack M, Klein NK, Taylor HG (1995). Long-term developmental outcomes of low birth weight infants. Future Child.

[11] Walhovd KB, Watts R, Amlien I, Woodward LJ (2012). Neural tract development of infants born to methadone-maintained mothers. Pediatr Neurol.

[12] Hunt RW, Tzioumi D, Collins E, Jeffery HE (2008). Adverse neurodevelopmental outcome of infants exposed to opiate in-utero. Early Hum Dev.

[13] Walhovd KB, Watts R, Amlien I, Woodward LJ (2012). Neural tract development of infants born to methadone-maintained mothers. Pediatr Neurol.

[14] McLemore GL, Lewis T, Jones CH, Gauda EB (2013). Novel pharmacotherapeutic strategies for treatment of opioid-induced neonatal abstinence syndrome. Semin Fetal Neonatal Med.

[15] Patrick SW, Schumacher RE, Benneyworth BD, Krans EE, McAllister JM (2012). Neonatal abstinence syndrome and associated health care expenditures United States, 2000–2009. Jama-Journal of the American Medical Association.

[16] Salehi M, Kheirabadi GR, Maracy MR, Ranjkesh M (2011). Importance of gabapentin dose in treatment of opioid withdrawal. J Clin Psychopharmacol.

[17] Zullino DF, Khazaal Y, Hättenschwiler J, Borgeat F, Besson J (2004). Anticonvulsant drugs in the treatment of substance withdrawal. Drugs Today (Barc).

[18] Kämmerer N, Lemenager T, Grosshans M, Kiefer F, Hermann D (2012). Pregabalin for the reduction of opiate withdrawal symptoms. Psychiatr Prax.

[19] Xi ZX, Stein EA (2000). Increased mesolimbic GABA concentration blocks heroin self-administration in the rat. J Pharmacol Exp Ther.

[20] Gerasimov MR, Dewey SL (1999). Gamma-vinyl gamma-aminobutyric acid attenuates the synergistic elevations of nucleus accumbens dopamine produced by a cocaine/heroin (speedball) challenge. Eur J Pharmacol.

[21] Brodie JD, Case BG, Figueroa E, Dewey SL, Robinson JA (2009). Randomized, double-blind, placebo-controlled trial of vigabatrin for the treatment of cocaine dependence in Mexican parolees. Am J Psychiatry.

[22] Brodie JD, Figueroa E, Dewey SL (2003). Treating cocaine addiction: From preclinical to clinical trial experience with gamma-vinyl GABA. Synapse.

[23] Brodie JD, Figueroa E, Laska EM, Dewey SL (2005). Safety and efficacy of gamma-vinyl GABA (GVG) for the treatment of methamphetamine and/or cocaine addiction. Synapse.

[24] Lawthom C, Smith PE, Wild JM (2009). In utero exposure to vigabatrin: No indication of visual field loss. Epilepsia.

[25] Sorri I, Herrgård E, Viinikainen K, Pääkkönen A, Heinonen S (2005). Ophthalmologic and neurologic findings in two children exposed to vigabatrin in utero. Epilepsy Res.

[26] Browne TR (1998). Pharmacokinetics of antiepileptic drugs. Neurology.

[27] Rey E, Pons G, Olive G (1992). Vigabatrin. Clinical pharmacokinetics. Clin Pharmacokinet.

[28] Agnish ND, Keller KA (1997). The rationale for culling of rodent litters. Fundam Appl Toxicol.

[29] Jones KL, Barr GA (2000). Opiate withdrawal in the fetal rat: A Behavioural profile. Pharmacol Biochem Behav.

[30] Richardson KA, Yohay AL, Gauda EB, McLemore GL (2006). Neonatal animal models of opiate withdrawal. ILAR J.

[31] Razali NM, Wah YB (2011). Power comparisons of shapiro-wilk, kolmogorov-smirnov, lilliefors and anderson-darling tests. J Stat Mod and Analytics.

[32] Shapiro SS, Wilk MB (1965). An analysis of variance test for normality (Complete Samples). Biometrika.

[33] Ghasemi A, Zahediasl S (2012). Normality tests for statistical analysis: a guide for non-statisticians. Int J Endocrinol Metab.

[34] Festing MF (2006). Design and statistical methods in studies using animal models of development. ILAR J.

[35] Lazic SE, Essioux L (2013). Improving basic and translational science by accounting for litter-to-litter variation in animal models. BMC Neurosci.

[36] Haseman JK, Hogan MD (1975). Selection of the experimental unit in teratology studies. Teratology.

[37] Marsh DF, Hatch DJ, Fitzgerald M (1997). Opioid systems and the newborn. Br J Anaesth.

[38] Abdulrazzaq YM, Padmanabhan R, Bastaki SM, Ibrahim A, Bener A (2001). Placental transfer of vigabatrin (gamma-vinyl GABA) and its effect on concentration of amino acids in the embryo of TO mice. Teratology.

[39] Center for Substance Abuse (2004). T. SAMHSA/CSAT Treatment Improvement Protocols. Clinical Guidelines for the Use of Buprenorphine in the Treatment of Opioid Addiction.

[40] Olmedo R, Hoffman RS (2000). Withdrawal syndromes. Emerg Med Clin North Am.

[41] Farrell M (1994). Opiate withdrawal. Addiction.

[42] Lynch WJ, Nicholson KL, Dance ME, Morgan RW, Foley PL (2010). Animal models of substance abuse and addiction: Implications for Science, Animal Welfare, and Society. Comp Med.

[43] Myers KM, Carlezon WA (2010). D-cycloserine facilitates extinction of naloxone-induced conditioned place aversion in morphine-dependent rats. Biol Psychiatry.

[44] Kanarek RB, D’Anci KE, Jurdak N, Mathes WF (2009). Running and addiction: precipitated withdrawal in a rat model of activity-based anorexia. Behav Neurosci.

[45] Smith LM, Chang L, Yonekura ML, Grob C, Osborn D (2001). Brain proton magnetic resonance spectroscopy in children exposed to methamphetamine in utero. Neurology.

[46] Smith LM, Chang L, Yonekura ML, Gilbride K, Kuo J (2001). Brain proton magnetic resonance spectroscopy and imaging in children exposed to cocaine in utero. Pediatrics.

[47] Tokuyama S, Takahashi M, Yamamoto T (2000). On the role of glutamate within the locus coeruleus during the development of opioid dependence and on the expression of withdrawal from dependence on opioids. Nihon Shinkei Seishin Yakurigaku Zasshi.

[48] Diaz SL, Barros VG, Antonelli MC, Rubio MC, Balerio GN (2006). Morphine withdrawal syndrome and its prevention with baclofen: Autoradiographic study of mu-opioid receptors in prepubertal male and female mice. Synapse.

[49] Rogawski MA, Löscher W (2004). The neurobiology of antiepileptic drugs for the treatment of non-epileptic conditions. Nat Med.

[50] Gram L, Larsson OM, Johnsen A, Schousboe A (1989). Experimental studies of the influence of vigabatrin on the GABA system. Br J Clin Pharmacol.

[51] Ashton H, Young AH (2003). GABA-ergic drugs: Exit stage left, enter stage right. J Psychopharmacol.

[52] Rundfeldt C, Löscher W (1992). Development of tolerance to the anticonvulsant effect of vigabatrin in amygdala-kindled rats. Eur J Pharmacol.

[53] Tyacke RJ, Lingford-Hughes A, Reed LJ, Nutt DJ (2010). GABAB receptors in addiction and its treatment. Adv Pharmacol.

